# Midwifery

**Published:** 1838-01

**Authors:** 


### MIDWIFERY.

*Cases of Inversion of the Uterus; with Remarks.* By Thomas Radford, Surgeon to the Manchester Lying-in Hospital, &c.

This is a valuable practical paper. The author takes a comprehensive view of the whole subject,—examines candidly the various opinions of preceding writers as to the causes, nature, and treatment of the affection,—and renders good reasons for his own. We can only find room for a few extracts relating to this latter part of the enquiry.

*Causes of Inversion.* "This accident has been attributed to causes purely mechanical, the uterus being unresisting, and passively obedient to their influence. The practice of pulling too early and violently at the funis, after the expulsion of the child, before the uterus has contracted, so as to detach and expel the placenta, has been generally considered as the cause of inversion; but we know that the accident happens before any force has been applied to the funis. . . . It has occurred when the patient had been delivered of a dead child, the funis so putrid as to break with a very slight effort. It has been found before the cord was separated, and the child given to the nurse. In the practice of Ruysch, this circumstance took place after he had extracted a dead child, &c. These circumstances shew that there is a power inherent in the uterus to become inverted. The pulling of the funis is so common a practice amongst our midwives, and done without the least consideration of the condition of the uterus, that, if it was so frequent a cause as is usually stated, inversion, instead of being one of the most rare, would be the most common accident in midwifery. Some writers have thought that a short funis is a frequent cause of inversion; whilst others think, in order to act, it must be inserted in the centre of the placenta, and that this mass must be attached to the fundus uteri. Now, it is evident, if brevity of the cord is capable of producing so serious an accident, these peculiarities will greatly add to its influence. But, amongst the published cases of inversion, there is, so far as the writer knows, but one where this shortness existed. It often occurs without diminished length in the cord; whilst, on the contrary, children are frequently born where it is very short, and yet no such event happens. The funis has been ruptured, and the placenta disrupted, and yet the uterus was not inverted.

"In order that the causes which have been now alluded to could operate effectually to produce inversion, there must be such condition of the uterus present that it becomes tacitly obedient to their influence. Most systematic writers, as also others, have supposed such to be the case. They have said that the uterus, previous to inversion, is in a state of extreme relaxation, exhaustion, or collapse, and that it offers no resistance to any force applied by the funis. These opinions are at variance with that of the writer.

"It appears to the writer that the uterine pain, diminution of bulk, firm resisting feel, sudden formation, and rapid protrusion, warrant him in the deduction that the *fundus* and *body* of the uterus, so far from being in a state of *collapse* or *relaxation*, are really in a state of *unnatural excitement* and *action*. But this is not the case with the os uteri: on the contrary, it is soft and yielding, as we find that it offers no resistance to the coming down of the tumour, whose protrusion is forcible and rapid. If these statements be true, it is evident that the fundus and os uteri are in directly opposite conditions: the former is in a state of violent contraction, the latter in a state of relaxation; and that this relative difference in these two parts of the organ is indispensably necessary to exist where inversion occurs.

"From what has been stated, it may be concluded that quick labour, whether natural or artificial,—a disturbance of this process in any of its stages,—or any of those circumstances which produce irregular contraction of the uterus, are, singly or combined, the causes of inversion.

*Treatment.* "When the uterus is inverted only in a slight degree, the reduction may be accomplished with great ease, and the attempt should be made as soon as it is discovered. As the fundus uteri has not, or only slightly, passed through the os, the placenta cannot wholly protrude through this orifice; and, consequently, the fundus should be returned before the placenta is separated. For, if an attempt were made to detach the placenta, the operation must be slow, uncertain, and incomplete, and the danger of hemorrhage incurred, or a greater degree of inversion produced. When the hand is introduced through the os uteri, the fingers should be slightly bent, so as to form a kind of crutch, to carry up the fundus, which sometimes rapidly springs up. The placenta is now to be separated, and the hand retained until the uterus contracts. . .

"In the treatment of this accident, [great inversion,] the great object to be constantly kept in view is to attempt the reinversion as soon as possible after the occurrence. But in general the placenta adheres to the inverted organ, and the question is whether it should be separated or not before or after the reduction. It is an important point to settle, especially as there is such a difference of opinion upon the subject. . . . The dread of hemorrhage is the reason assigned why the placenta should not be first detached, but the writer trusts that the cases he has adduced, and the references he has made, are sufficient evidence to the contrary. In no case has this dreaded effect been induced, or even aggravated, by a *complete* separation of the placenta. The uterine vessels are as effectually constricted under this accident as when the organ is in its natural situation, if the placenta be entirely detached; and flooding is produced here, in the same manner as in ordinary cases, by a partial separation or disruption. As the greatest disadvantage arises from failing in our first attempt, it is the more necessary that every impediment should be removed, so that we can proceed with the greatest chance of success. By delay, the organ becomes less fit to bear the operation, not only from the increased size of the fundus and the contraction of the os, but also from the increased sensibility and irritability which it has acquired, even previously to its becoming actually inflamed. The attached placenta must increase the obstacle, because the fundus cannot be so freely and sufficiently compressed. The result of free manipulation would lead to partial detachment and disruption, and consequently to flooding. By detaching the placenta, great advantages are gained; the bulk of the part is diminished, the operator is enabled further to reduce the size of the fundus itself by compression; and he has more freedom to judge of the changes he has effected. . . .

"When the placenta is detached, our next object should be to attempt the reduction of the general bulk of the tumour, by compressing it. We are indebted to Mr. C. White for this method. The plan recommended by some writers, to push the fundus directly upwards, should not be adopted. There are strong reasons to think that the fundus is, after the os uteri, the most irritable part of this organ. When the accident has existed a short time, pressure upon this portion induces pain, bearing down, and hemorrhage; but the body may be taken hold of, and compressed. If we could press the fundus upwards, and thereby dimple it within itself, we should find ourselves opposed by a double inflexion; for the body would be grasped by the os uteri, and the fundus would be within the body. It is obvious that our force should be directed so as to act upon the angle of inflexion, or where it turns into itself. . . .

"It will be found that the tumour will freely pass through the os externum, and, as only one hand can be admitted into the vagina, the chief compression should be effected whilst it lies externally. And, as the upper part of the vagina descends along with the uterus, no real effect can be produced until it is made tense by carrying this organ upwards. When it arrives at this point, resistance is met with; but, by keeping a steady pressure upwards, the inflected portion of the cervix then yields, and it gradually recedes, followed by the hand of the operator, until the reduction is completed." *Dublin Journal. Sept. and Nov.* 1837.

---

*Case of Hidrosis, or Hidrotic Fevers, with Remarks.* By J. C. W. Lever, Surgeon.

This is a minutely described and very interesting case of one of the many varieties of affections which are usually confounded under the general name of Puerperal Fever. It has received the above name from Dr. Blundell, owing, we presume, to the continued and profuse perspirations which constitute a marked feature in this variety. We cannot agree with either Dr. Blundell or Mr. Lever in regarding this disease as distinct from puerperal fever, until we find pathologists more agreed as to what puerperal fever really is. At any rate, the affection here described by Mr. Lever is well known to all experienced practitioners as one of the most untractable forms of the puerperal malady. It has been well and very particularly described by Dr. Marshall Hall, many years since. In this case the patient, as frequently occurs, was in a state of disorder and general irritation long before her confinement, insomuch that the fatal result might have been prognosticated. "During the last three months of gestation, she suffered from sickness, from a constant dull pain in her left side, in front, near the linea semilunaris, midway between the crest of the ilium and the extremities of the floating ribs,—from relaxation of the bowels, frequently having as many as four or five motions in the course of twenty-four hours,—and from almost perpetual watchfulness, so that night after night was passed without one wink of sleep." The pulse was also at this time extremely variable and quick, ranging from 100 to 150: and it retained this character, in an increased degree, throughout the course of the disease. The other most marked symptoms were, the continual profuse colliquative sweats; diminution, but not entire suppression, of the secretion of milk; copious secretion of urine; extreme irritability of the nervous system, with great mental excitement or forced calmness; abdominal tenderness; tympanitis and diarrhœa; tongue clean. The patient died on the eighteenth day after delivery. The only morbid appearances of importance were those in the uterus. "On the left side of the uterus, where the placenta was adherent, and where the pain and soreness were located, only coagula were found occupying the uterine sinuses and veins, such as are commonly met with when the uterus is examined a few days after delivery, while on the right side, where there was no pain nor even uneasiness, there were spots of ecchymosis, ulceration, and softening; the uterine sinuses and veins were even found filled with a fluid more or less purulent, corresponding in its colour and nature with the discharge which was received on the napkins during life." The treatment employed in this case was various, (perhaps too varied,) and consisted of aperients, opiates, mercury, quinine, astringents, &c. Cases of this kind often resist all treatment, even the most rational.

*Med. Gazette. Sept.* 9, 1837.

